# Factors influencing self-quantification for patients with hypertension: A cross-sectional Study

**DOI:** 10.1097/MD.0000000000036185

**Published:** 2023-12-01

**Authors:** Guiyue Ma, Haiyan Fang, Xiang Wang, Yahui Meng, Yu Zhu, Chuanying Zhang

**Affiliations:** a Nursing College, Anhui University of Chinese Medicine, Hefei, China.

**Keywords:** factor, hypertension, self-management, self-quantification

## Abstract

This study aimed to investigate the level of self-quantification among patients with hypertension and identify the factors influencing this behavior. This study aimed to investigate self-quantification levels and identify influencing factors among 400 patients diagnosed with hypertension. Employing a convenience sampling method, the research was conducted across diverse healthcare settings, including a tertiary hospital, 2 community hospitals, 2 pension institutions, and 5 residential areas. Participants underwent assessment using a self-quantification scale. The collected data underwent thorough analysis using various statistical methods, including descriptive analysis for an overview, 2 independent samples *t* test for mean comparisons, one-way analysis of variance for variations among groups, and multiple linear regression analysis to identify influential factors. This robust methodology was applied to gain comprehensive insights into the self-quantification behaviors of patients with hypertension. The total self-quantification score for patients with hypertension was found to be (96.64 ± 14.16). The average value for all dimensions was (3.22 ± 0.47). Notably, medical insurance type, education level, age, and complications were identified as significant factors influencing self-quantification among patients with hypertension. The study concludes that patients without medical insurance, with lower education levels, older age, and no complications tended to have lower levels of self-quantification. These findings underscore the necessity for targeted interventions to improve self-quantification in these specific patient groups. By addressing the identified influencing factors, healthcare providers can implement measures to enhance self-quantification among patients with hypertension.

## 1. Introduction

Hypertension is a major risk factor for cardiovascular disease (CVD) globally, impacting a significant number of individuals, with approximately 1.39 billion people affected. Elevated blood pressure is a leading cause of preventable CVD-related mortality and disease burden worldwide, spanning various regions.^[1]^ A comprehensive systematic review, which covered studies from 90 countries, revealed that the prevalence of hypertension stands at 31.1%. Notably, low- and middle-income countries exhibited a higher prevalence of hypertension (31.5%) compared to high-income countries (28.5%). This highlights the importance of addressing hypertension, particularly in regions with limited resources, to reduce its impact on CVD rates and improve global health outcomes.^[2]^ In China, hypertension has a significant prevalence, affecting approximately 63.7% of the population (58.3% in males and 69.0% in females). However, awareness about hypertension remains relatively low, with only 42.4% of individuals being aware of their condition (35.7% in males and 48.0% in females). The treatment rate for hypertension is 38.2%, with a higher rate in females (43.3%) compared to males (32.0%). Unfortunately, the control rate for hypertension is relatively low, with only 9.0% of individuals having their blood pressure under control (8.1% in males and 9.7% in females).^[3]^ These findings indicate a need for improved awareness, treatment, and control measures to effectively manage hypertension and reduce its impact on public health in China. All guidelines stress the importance of patient adherence to maintain blood pressure control.

In China, the healthcare system faces challenges characterized by high demand for medical services and uneven distribution of medical resources, resulting in a significant burden of out-of-pocket payments for patients. This situation has prompted a realization among the public that active participation in disease self-management is a crucial and effective approach to reduce morbidity and safeguard health.^[4]^ With the recognition of the limitations in the current medical service model, individuals are increasingly understanding the importance of taking control of their health through self-management practices.

The rise of wearable devices has led to increased attention towards self-quantification, and its application in the self-management of patients with hypertension has been steadily growing. Despite its significance as a healthcare trend, there remains a lack of comprehensive studies on the behavior of patients with hypertension concerning self-quantification.^[5]^ In order to improve and innovate self-quantification services, and to avoid the inefficient allocation of medical resources by overlooking crucial factors relevant to patients with hypertension, a deeper understanding of their usage behavior becomes imperative. Gaining insights into how patients with hypertension engage with self-quantification tools will facilitate the development of more effective and targeted interventions, ultimately leading to improved disease management and better health outcomes.

The concept of self-quantification was initially introduced by Gary Wolf and Kevin Kelly, editors of Wired magazine. They defined self-quantification as the utilization of technology and devices to monitor and investigate the human body. This practice, also referred to as “self-monitoring,” “self-tracking,” “personal analysis,” or “life hacker,” involves collecting and recording data to track an individual’s behavior and health metrics.^[6]^ In the context of hypertension, self-quantification pertains to the use of technology and devices to record, track, and quantify indicators related to high blood pressure. Through data feedback, individuals can engage in self-adjustment and self-management to effectively address their hypertension condition. This approach empowers patients to take a more active role in their health management, leading to better control of their blood pressure and improved overall well-being.^[7]^

Self-quantification is an emerging self-management approach in healthcare, distinguished by its precision, personalization, visualization, and intelligence. This method has found application in hypertension tracking and monitoring, gradually transforming the traditional health management paradigm. With self-quantification, individuals are empowered to take charge of their health by relying less on medical staff and more on themselves. Through active participation in self-health management, people can leverage the benefits of data-driven insights and make informed decisions to improve their well-being and effectively manage hypertension. This shift towards self-empowerment is revolutionizing the way healthcare is approached, fostering a more proactive and engaged approach to personal health.^[8]^

Effective self-management is particularly crucial for patients with hypertension. By employing smart devices and self-quantification techniques, patients with hypertension can continuously and non-invasively monitor their vital signs and behavioral information at any time and from anywhere.^[9]^ This ongoing monitoring aids in tracking disease progress and is beneficial for self-management of their condition. Research has demonstrated that self-quantification can lead to various positive outcomes for patients with hypertension.^[10]^ It has been proven to reduce blood pressure levels, enhance medication compliance,^[11]^ and decrease the frequency of medical consultations without increasing overall healthcare costs.^[12]^ By actively participating in self-quantification and self-management, patients can take greater control of their health, leading to improved health outcomes and a more efficient use of healthcare resources.

Limited research exists on the status of self-quantification among patients with hypertension, leaving a gap in understanding its potential impact on self-management. To address this gap and explore how patients with hypertension can effectively utilize self-quantification for better self-management and collaborative health management with medical professionals, we conducted a cross-sectional study in XX, XX, China. The primary objective of this study was to assess the level of self-quantification among a group of Chinese patients with hypertension. Additionally, we aimed to identify significant determinants that influence the adoption and effectiveness of self-quantification in this cohort. By shedding light on these aspects, the research seeks to contribute valuable insights into the potential benefits and challenges of self-quantification as a tool for enhancing self-management in patients with hypertension. The findings will hopefully support the development of more targeted and effective interventions to improve the overall health outcomes of patients with hypertension in China.

## 2. Method

### 2.1. Design and setting

A cross-sectional study with a convenience sampling and questionnaire survey is used to investigate the current situation of self-quantification among patients with hypertension. Guidelines for reporting results using observational descriptive studies (STROBE Statement) checklist were used. The study was conducted in Hunan, China, from December 2020 to January 2021.

### 2.2. Study sample

Participants for this study were selected using a convenience sampling method from various healthcare settings, including a tertiary hospital, 2 community hospitals, 2 pension institutions, and 5 residential areas. The inclusion criteria for the study were as follows: meeting the diagnostic criteria for essential hypertension based on the International Society of Hypertension 2020 International Guidelines for Hypertension Practice, with a systolic blood pressure of ≥140 mm Hg and/or a diastolic blood pressure of ≥90 mm Hg^[13]^; being 18 years of age or older; having the ability to communicate in and understand the Chinese language and expressing voluntary agreement to participate in the study; and demonstrating the ability to comprehend the study’s purpose and the content of the questionnaire. On the other hand, participants were excluded from the study if they met any of the following criteria: having hypertensive crisis, hypertensive encephalopathy, or acute hypertension; presenting with other major chronic diseases such as chronic obstructive emphysema, severe hepatic or renal insufficiency, or advanced malignant tumors; and suffering from mental instability or severe mental disorders. The sample size was determined reaching 15 to 20 times the number of selected variables. In this study, a total of 13 demographic were considered independent variables, so at least 195 to 260 participants were needed. Considering 20% inefficiency, meaning that the sample size was at least 234 to 312. In total, we planned to recruit 400 participants.

### 2.3. Data collection

To ensure the accuracy and reliability of the research, the research assistants underwent training to understand the research purpose, content, and ethical considerations before conducting the investigation. Data collection took place in a designated meeting room. During the data collection process, the research assistant explained the research procedures and objectives to the participants and obtained their informed consent. The informed consent covered details about the participants’ right to withdraw from the study at any time and ensured the anonymity and confidentiality of their information. To participate, individuals were required to complete a self-reported questionnaire. To further protect the participants’ privacy, each participant was assigned a unique serial number, and the collected data were securely stored in a locked box to maintain confidentiality. These measures were put in place to uphold ethical standards and safeguard the well-being and rights of the study participants throughout the research process. It took about 15 to 20 minutes to complete the questionnaire.

### 2.4. Measurements

#### 2.4.1. General information.

A self-developed questionnaire was utilized to gather demographic information from the participants, encompassing details such as gender, age, marital status, education level, and occupation, family per capita monthly income, family place of residence, medical insurance, course of illness, complications, types and quantity of medication, family history.

#### 2.4.2. Self-quantification Scale for Patients with Hypertension.

Ma et al^[7]^ developed the Self-Quantification Scale for Patients with Hypertension. This scale comprises 30 items, organized into 7 dimensions: external reward (3 items), internal reward (5 items), severity (4 items), susceptibility (5 items), response efficacy (5 items), self-efficacy (5 items), and response cost (3 items). Respondents rate their agreement on a 5-point Likert scale, with the response cost dimension scored in reverse. The scale’s total scores range from 30 to 150 points, where higher scores indicate greater levels of self-quantification. In this study, the researchers assessed the reliability and validity of the scale. Cronbach’s α coefficient, split-half coefficient, and test-retest reliability coefficient were found to be 0.900, 0.743, and 0.880, respectively. These results demonstrated good reliability and validity of the Self-Quantification Scale for Patients with Hypertension, indicating its suitability for assessing self-quantification behavior in the context of hypertension management.

### 2.5. Data analysis

Data analysis was performed using SPSS software version 27.0. Descriptive statistics were used to present the demographic characteristics of the participants. The level of self-quantification among patients was described using mean values, standard deviations, and percentages. Frequencies and percentages were employed to summarize the results. For the analysis of continuous data, *t* tests and ANOVA were utilized. *T* tests were conducted for univariate analysis, while multiple linear regression was employed to identify the determinants of self-quantification. Statistical significance was determined using two-tailed tests, and a significance level of *P* < .05 was considered statistically significant.

### 2.6. Ethical considerations

The study received ethical approval from Central South University in China before its initiation (E202071). Participants were informed that their involvement in the study was entirely voluntary, and they had the freedom to decide whether or not to participate. To ensure the privacy and confidentiality of the participants, the questionnaires used in the study were designed to be completely anonymous. These measures were implemented to uphold ethical standards and protect the rights and well-being of the study participants throughout the research process.

## 3. Results

### 3.1. General information

A total of 400 questionnaires were distributed for this study, out of which 390 were deemed valid, resulting in an effective response rate of 97.5%. The demographic characteristics of the participants are summarized in Table 1. The majority of the study population were women, constituting 53.6% of the total respondents.

**Table 1:** Socio-demographic characteristics of participants (N = 390).

Item		n	%
Gender	Male	181	46.4
	Female	209	53.6
Age	18–30	3	0.8
	31–40	7	1.8
	41–50	41	10.5
	51–60	96	24.6
	61–70	156	40.0
	≥70	87	22.3
Marital status	Unmarried	2	0.5
	Married	331	84.9
	Widow	49	12.6
	Divorce	8	2.1
Education level	Primary school or below	140	35.9
	Junior middle school	144	36.9
	High school	67	17.2
	Bachelor degree or above	39	10.0
Occupation	Jobless	17	4.4
	Farming	8	2.1
	Occupied	122	31.3
	Retired	243	62.3
Monthly per capita household income	≤1000	129	33.1
	1000–3000	140	35.9
	3001–5000	101	25.9
	≥5000	20	5.1
Habitation	City	321	82.3
	Rural	69	17.7
Living situation	Live alone	33	8.5
	Live with your family	357	91.5
Type of medical insurance	Medical insurance for urban residents	188	48.2
	New rural cooperative medical insurance	190	48.7
	Without medical insurance	12	3.1
A history of hypertension (years)	≤1	10	2.6
	1–5	75	19.2
	6–10	86	22.1
	≥10	219	56.2
Complication	No	144	36.9
	Yes	246	63.1
Different types of antihypertensive medications	1	330	84.6
	2	49	12.6
	≥3	11	2.8
Family history of hypertension	No	174	44.6
	Yes	216	55.4

### 3.2. Scores of each dimension of self-quantification for patients with hypertension

Table 2 presents the scores for each dimension of self-quantification among patients with hypertension. The average value for all dimensions is (3.22 ± 0.47). Among them, the average score of severity was the highest (3.50 ± 0.73), the average score ranging from high to low is the response cost, internal reward, self-efficacy response efficiency, and susceptibility, while the score of external reward was the lowest (2.61 ± 1.33).

**Table 2:** Scores of each dimension of self-quantification of patients with hypertension (N = 390).

Item	Total score (*X̅* ± *S*)	Average score (*X̅* ± *S*)
External reward	7.82 ± 3.99	2.61 ± 1.33
Internal reward	16.98 ± 3.04	3.40 ± 0.61
Severity	13.99 ± 2.90	3.50 ± 0.73
Susceptibility	15.08 ± 3.91	3.02 ± 0.78
Response efficiency	16.08 ± 3.04	3.22 ± 0.61
Self-efficacy	16.35 ± 5.12	3.27 ± 1.02
Response cost	10.33 ± 3.50	3.44 ± 1.17
Total score	96.64 ± 14.16	3.22 ± 0.47

### 3.3. Scores on the self-quantification scale for patients with hypertension varied based on different characteristics

As shown in Table 3. The results indicated that self-quantification among patients with hypertension exhibited statistically significant differences concerning age, marital status, education level, occupation, monthly per capita household income, habitation, living situation, type of medical insurance, history of hypertension, and complications (*P* < .05). In summary, the study found that various demographic and health-related factors significantly influenced the level of self-quantification among patients with hypertension.

**Table 3:** Scores on self-quantification for patients with hypertension with different characteristics (N = 390).

Item		*X̅* ± *S*	*t*/*F*	*P*
Gender	Male	96.17 ± 14.81	−0.616	.538
	Female	97.06 ± 13.59		
Age	18–30	132.67 ± 6.03	37.700	.000
	31–40	108.14 ± 10.71		
	41–50	109.83 ± 7.48		
	51–60	102.68 ± 9.30		
	61–70	93.96 ± 13.12		
	≥70	86.41 ± 12.93		
Marital status	Unmarried	85.50 ± 2.12	51.235	.000
	Married	99.68 ± 12.25		
	Widow	77.04 ± 11.17		
	Divorce	93.75 ± 6.02		
Education level	Primary school or below	89.16 ± 14.48	41.244	.000
	Junior middle school	97.09 ± 11.58		
	High school	102.64 ± 9.74		
	Bachelor degree or above	111.54 ± 10.84		
Occupation	Jobless	97.71 ± 6.89	44.364	.000
	Farming	102.88 ± 11.75		
	Occupied	106.81 ± 9.95		
	Retired	91.26 ± 13.52		
Monthly per capita household income	≤1000	91.25 ± 13.93	20.943	.000
	1000–3000	95.69 ± 13.04		
	3001–5000	102.01 ± 12.33		
	≥5000	111.00 ± 13.29		
Habitation	City	98.79 ± 12.58	5.689	.000
	Rural	86.67 ± 16.71		
Living situation	Live alone	81.61 ± 14.94	−6.731	.000
	Live with your family	98.03 ± 13.27		
Type of medical insurance	Medical insurance for urban residents	101.14 ± 12.21	27.044	.000
	New rural cooperative medical insurance	93.29 ± 13.81		
	Without medical insurance	79.25 ± 20.21		
A history of hypertension (years)	≤1	99.20 ± 12.34	3.933	.009
	1–5	100.55 ± 12.18		
	6–10	98.14 ± 14.00		
	≥10	94.60 ± 14.63		
Complication	No	94.23 ± 14.83	−2.596	.010
	Yes	98.06 ± 13.58		
Type of medication	1	96.38 ± 13.97	0.367	.693
	2	98.12 ± 14.80		
	≥3	97.91 ± 17.49		
Family history of hypertension	No	96.91 ± 14.42	0.338	.736
	Yes	96.43 ± 13.97		

### 3.4. Multiple linear regression analysis on influencing factors of self-quantification for patients with hypertension

In this study, multiple linear regression analysis was conducted to identify the influencing factors of self-quantification among patients with hypertension. The dependent variables included the overall score of self-quantification and the scores of each dimension of self-quantification. The independent variables considered were age, marital status, education level, occupation, monthly per capita household income, habitation, living situation, type of medical insurance, history of hypertension, and complications (specific assignments are detailed in Table 4). The results of the multiple linear regression analysis revealed that the main influencing factors of self-quantification for patients with hypertension were the type of medical insurance, education level, age, and the presence of complications. These factors had a statistically significant impact on the level of self-quantification among patients with hypertension. Among them, the main influencing factors of external reward dimension are education level and complications; the influencing factors of internal reward dimension are type of medical insurance, age and education level; the main influencing factors of severity dimension are medical insurance type, age, education level and living situation; the main influencing factors of susceptibility dimension are age, family location, education level and complications; the main influencing factors of response efficacy dimension are medical insurance type, education level and age. Table 5 reveals the main influencing factors for each dimension of self-quantification. For the self-efficacy dimension, the primary influencing factors are occupation, type of medical insurance, education level, and complications. On the other hand, for the response cost dimension, the main influencing factors are occupation, type of medical insurance, education level, living situation, and age.

**Table 4:** Variables assignment of analysis on influencing factors of self-quantification for patients with hypertension.

Variables	Assignment
Age	18–30 = 1; 31–40 = 2; 41–50 = 3; 51–60 = 4; 61–70 = 5; ≥70 = 6
Marital status	Marital status referring to “unmarried” as dummy variable: M1 = married (1, 0, 0); M2 = widowed (0, 1, 0); M3 = divorced (0, 0, 1) Degree of education primary
Education level	Primary school and below = 1; junior middle school = 2; high school = 3; bachelor degree or above = 4
Occupation	Referring to the jobless as dummy variable: P1 = farming (1, 0, 0); P2 = Occupied; (0, 1, 0); P3 = Retired (0, 0, 1)
Monthly per capita household income	≤1000 = 1; 1000–3000 = 2; 3001–5000 = 3; ≥5000 = 4
Habitation	Urban area = 1; rural area = 2
Living situation	Live alone = 1; live with your family = 2
Type of medical insurance	Referring to “medical insurance for urban residents” as dummy variable: I1 = new rural cooperative medical insurance (0, 1, 0); I2 = without medical insurance (0, 0, 1)
A history of hypertension (yr)	≤1 = 1; 1–5 = 2; 6–10 = 3; ≥10 = 4
Complication	No = 0; Yes = 1
External reward	Actual value, continuous variable
Internal reward	Actual value, continuous variable
Severity	Actual value, continuous variable
Susceptibility	Actual value, continuous variable
Response efficiency	Actual value, continuous variable
Self-efficacy	Actual value, continuous variable
Response cost	Actual value, continuous variable
Total score	Actual value, continuous variable

**Table 5:** Results of multiple linear regression analysis on the overall self-quantification for patients with hypertension (N = 390).

Model	*B*	SE	β	*T*	*P*
(constant)	102.871	8.804	–	11.685	.000*
Married	9.404	6.918	0.238	1.359	.175
Divorce	−3.383	7.022	−0.079	−0.482	.630
Widow	1.202	7.820	0.012	0.154	.878
Farming	1.853	4.458	0.019	0.416	.678
Occupied	−0.735	2.736	−0.024	−0.269	.788
retired	−3.738	2.871	−0.128	−1.302	.194
New rural cooperative medical insurance	−0.006	1.357	0.000	−0.004	.997
Without medical insurance	−10.071	3.011	−0.123	−3.345	.001*
Education level	3.491	0.735	0.238	4.749	.000*
Age	−3.511	0.921	−0.257	−3.811	.000*
Complication	3.831	1.035	0.131	3.703	.000*

* *P* < .05.

## 4. Discussion

### 4.1. Improving self-quantification level for patients with hypertension

Currently, no studies have investigated the influencing factors related to self-quantification in patients with hypertension. However, self-quantification management plays a crucial role in hypertension prevention and control. This study aims to examine the current status of self-quantifi12cation among patients with hypertension in China and identify the related influencing factors. The findings revealed that the overall self-quantification score was (96.64 ± 14.16). The average value for all dimensions is (3.22 ± 0.47). The results indicate that self-quantification among patients with hypertension is not at a high level. Therefore, it is essential to explore the factors influencing self-quantification in this population to enhance their self-management practices.

### 4.2. Influencing factors on self-quantification level in patients with hypertension: the role of medical insurance

The study findings indicated that the type of medical insurance significantly influenced the self-quantification level among patients with hypertension, with those without medical insurance having lower total scores. One possible reason for this observation is that patients covered by urban residents’ medical insurance tend to have better economic conditions and higher awareness of smart devices for blood pressure management. Consequently, they encounter fewer difficulties in using such devices. These results align with previous research conducted by Guo et al.^[14]^ The income of patients without medical insurance is mainly used for basic living and medical treatment, and it is difficult to afford the extra cost to manage their own health. Hence, it is crucial to emphasize the advantages of self-quantification in health management to patients without medical insurance. Encouraging them to adopt self-quantification practices can help in timely prevention and control of cardiovascular events, effectively safeguarding their health and well-being.

### 4.3. Influence of education level on self-quantification level in patients with hypertension

Study findings revealed a positive correlation between the level of education and the total self-quantification score among patients with hypertension. Specifically, higher levels of education were associated with greater self-quantification levels, indicating that individuals with higher education tend to engage more actively in self-management practices for hypertension. Patients with high level of education are more receptive to the way of regularly understanding physical changes and actively obtaining relevant data through smart devices, and their cognitive model has gradually changed from general experience-driven to data-driven quantitative cognitive model.^[15]^ They are also more rational and scientific in analyzing problems. And patients with a high level of education are more likely to use smart devices, which is conducive to the improvement of self-quantification. In addition, it has been found that patients’ blood pressure is poorly controlled because of their low education level, which leads to low self-control of the disease.^[16]^ This result shows that those with higher education have higher awareness of self-management to improve health, leading patients to effective self-management. Therefore, for patients with low educational level, their motivation for self-quantification needs to be improved from a cognitive perspective, thus promoting the improvement of their self-quantification.

### 4.4. Age influencing on level of self-quantification for patients with hypertension

The study findings indicated a positive relationship between younger age and higher total scores on self-quantification. In other words, younger individuals tended to exhibit higher levels of self-quantification, suggesting that they were more actively involved in managing their hypertension using self-monitoring and other technological tools. The study showed 243 million people over the age of 60 nationwide, with only 23.2% of them using online.^[17]^ Compared with older people, younger people are easier to use smart devices and accept self-quantification. Middle-aged and elderly patients lack awareness of the benefits of self-quantification due to the increase of age.^[18]^ The prevention and treatment of hypertension cannot only rely on antihypertensive drugs to control.^[19]^ The elderly improve motivation and conducive to improving their ability of self-management.^[20]^ However, for older people, fewer people use smart devices. Because patients have an unknown understanding of smart devices, they worry about the accuracy of smart devices is not enough and difficult to use. Therefore, for elderly patients with hypertension, it is necessary to introduce the use and function of smart devices in detail, so that they are willing to use smart devices for self-quantification management.

### 4.5. Complication influencing on level of self-quantification for patients with hypertension

The study results indicated that the presence of comorbidities did influence the level of self-quantification among patients with hypertension. The findings suggest that patients with additional health conditions may be more motivated to engage in self-quantification practices, likely due to the increased awareness of the importance of managing multiple health conditions and the need for vigilant self-monitoring. Generally speaking, patients with complications know more patients, and their relatives and friends will encourage them to try more ways to improve their health, and the motivation of the patients will increase. Patients with complications have higher ability of self-management and are more confident in using smart devices to manage blood pressure.^[21]^ Patients with hypertension combined with other diseases have poorer physical fitness and suffer more physical pain. So, they are more eager to manage their own health. Therefore, it is necessary to educate and actively guide patients with hypertension without complications, so as to produce self-quantification, which is conducive to better management of their own health.

### 4.6. Strengths and limitations

Our study boasts several notable strengths. Firstly, the data collected underwent analysis using robust statistical methods, ensuring the accuracy and validity of the results. Secondly, the analysis yielded easily interpretable findings, offering clear insights into the research subject. However, we acknowledge certain limitations in our study. Firstly, the sample size was relatively small, comprising participants from a single district in China. Thus, the generalizability of these findings to other regions may be limited, necessitating further research with larger and more diverse samples. Secondly, the same tool was utilized to assess participants of various ages, but its suitability may vary across different age groups, warranting caution in interpreting the results for specific age demographics. Finally, we only investigated demographic data and did not consider clinical characteristics. In the future, it will be important to also explore some clinical characteristics of the study sample.

## 5. Conclusion

The overall self-quantification level in patients with hypertension, as indicated by both the total and average scores, requires improvement. The study found that certain factors, including the type of medical insurance, education level, age, and the presence of complications, significantly influenced the level of self-quantification in these patients. Specifically, lower self-quantification levels were observed in patients without medical insurance, those with lower educational attainment, older individuals, and those without any complications. Addressing these influencing factors can help enhance self-quantification practices among patients with hypertension and improve their overall disease management and health outcomes.

## Acknowledgments

We acknowledge all the participants and assisting researchers.

## Author contributions

**Conceptualization:** Guiyue Ma.

**Data curation:** Guiyue Ma.

**Formal analysis:** Guiyue Ma.

**Funding acquisition:** Guiyue Ma.

**Investigation:** Guiyue Ma.

**Methodology:** Guiyue Ma.

**Project administration:** Guiyue Ma.

**Resources:** Guiyue Ma.

**Software:** Yu Zhu.

**Validation:** Guiyue Ma, Yahui Meng.

**Visualization:** Guiyue Ma, Yahui Meng, Yu Zhu.

**Writing – original draft:** Guiyue Ma.

**Writing – review & editing:** Haiyan Fang, Xiang Wang, Chuanying Zhang.
